# Case of Typhoid Fever Followed by Malignant Intermittent—Recovery—Employment of Quinine in Large Doses

**Published:** 1842-12-24

**Authors:** J. W. Lisle

**Affiliations:** Resident Physician


*Philadelphia Hospital, Blockley.—Service of Dr. Gerhard.*

Reported by J. W. Lisle, M. D., Resident Physician.

*Case of Typhoid Fever followed by Malignant Intermittent—Recovery—Employment of Quinine in large doses.*

Patrick Callaghan, æt. 26, an Irishman by birth, but for the last five years a resident of this country, has been subject to attacks of pain in left breast for the last six years, first occurring after long continued exposure to wet and damp, these attacks generally lasted about two weeks, and were attended with a troublesome cough, sensation of "smothering," and palpitation of heart. According to his own account, all remedies seemed to augment the symptoms, which, on being allowed to take their own course, subsided spontaneously. For three months preceding his entry into the Hospital, he was employed as a boatman on the Schuylkill, between Pottsville and Philadelphia, and while acting in that capacity was seized about the 24th September with a chill, accompanied by headache, pain in back, ringing in ears, pain in breast, and cough, with palpitation of heart; had three chills the day of attack, and on the following day symptoms of fever supervened, with great thirst, heat of skin, anorexia, pain in head, frequent inclination to go to stool, discharges thin, with griping pain; he was relieved by a dose of oil. The oppressive cough, cephalalgia, ringing in ears, &c., have continued up to the time of entering the Hospital, and have been attended with great restlessness and want of sleep.

Entered the Hospital on the morning of October 7th, 1842, presenting the following symptoms:

Face flushed; intellect clear; respiration hurried, and laboured, 28; abdomen full and tender; pulse 96, quick and feeble; a few sudamina on abdomen, but no red papulæ, no cephalalgia, no tinnitus aurium; had three or four stools yesterday, but none to day. Percussion good over chest in every part, excepting a slight dulness over lower part of left lung anteriorly. Auscultation—sibilant and sonorous rhonchus, throughout left lung anteriorly, mingled at lower portion with subcrepitant; sonorous rhonchus throughout left lung anteriorly; same sounds posteriorly, but less marked. Action of heart slightly increased. Sounds natural, rhythm perfect.

Oct. 7—Evening. Skin hot and dry, tongue smooth, red and moist, epigastrium very tender; had two stools during the day. Ordered surface to be sponged with tepid acidulated water, and six cut cups, three to epigastrium and three between the shoulders; barley water; low diet.

October 8. Relieved by the cups, which drew nine ounces of blood; slept very little. Perspired freely for about an hour; skin of moderate temperature; no stool since last visit; cough slight; dyspnœa continues; very little sibilant and sonorous ronchus; some mucous and subcrepitant on left side anteriorly.

℞. Mist. Neutralis.

Evening. Hot and dry skin, and every symptom of considerable fever; tongue moist; eyes injected, epigastric tenderness greater than in the morning, though he says less than usual; ordered castor oil ℥i.

October 9. No stool; fever moderated; pulse 112; slept very indifferently; skin hot and dry; red papulæ disappearing; epigastric tenderness continues.

℞ Oily Mixture during the day.

October 10. Complains of extreme weakness; profuse sweating an hour before visit; skin is now cool; somnolence slight; no stool from the oil until aided by injection, since which had two stools; slight flush of face; pulse one hundred, soft and undulating. Respiration thirty, rather high; tongue very red, clean and moist; no abdominal tenderness, except a little at epigastrium; few sudamina, no spots; tympanitis; considerable præcordial pain; action of heart feeble; sounds distant.

Respiration anteriorly on the left side very feeble, with sibilant and sonorous rhonchus; right side much more distinct. Posteriorly, similar evidences of slight bronchitis. Ordered cut cups iv. to left side posteriorly.

October 11. Considerably relieved by cups yesterday; slept badly last night; skin warm and dry; pulse 108, feeble, no subsultus; no red spots visible; auscultation same as at last visit; no stool since yesterday morning.

℞. Ol. Ricini, ℥j. q. h. iij.

℞. Pil. Hydrargyri, gr. j. ter in die.

October 12. Pulse soft, undulating, about 95; skin moist; expression calm; no subsultus; no ringing in ears; some epigastric uneasiness; much tympanitis; papulæ barely visible; some sudamina; slept well last night; tongue red and dry; bowels still costive; ordered a full dose of castor oil.

October 13. Complete apyrexia; skin of pleasant temperature; perspirable; less dyspnœa; bowels opened freely by the oil.

October 14. Sleep broken during the night; dozes through the day; skin of pleasant temperature; countenance characteristic. Pulse 100, feeble; bisferiens; no stool since yesterday morning; slight soreness of mouth. Discontinued the blue mass.

October 19. Symptoms have varied slowly since the 14th instant. A gradual improvement has taken place under a mild treatment, consisting of cooling drinks, tepid ablutions, and gentle laxatives; the insomnolence has continued more or less to the present time: considerable epistaxis last evening, followed by a refreshing sleep; costive; skin this morning moderately warm, pleasant; pulse 106. Ordered a laxative of castor oil.

October 23. On visiting him this morning found him considerably weaker, and in profuse perspiration. He was taken early last evening with chilliness and rigour, which lasted for an hour.

℞. Infus. Cinchonæ, ℥ij. q. h. iij.

October 24. Two chills since last visit, both of which were followed by exhausting sweats. He was ordered to take at once, Quin. Sulph. grs. x. Tinct. Opii. gtts. xx., and to be followed by doses of the same tonic of gr. v. each, every hour for eight hours; and then two more doses of gr. v. each, every second hour.

October 25. A slight chill was noticed yesterday after he had taken the fourth dose of quinine, but its duration was short—no return of the chill since—complains this morning of pain in head; has no pain in bowels; no purging; little or no epigastric tenderness.

Evening. Epigastrium very tender. Tongue red and dry; pulse quick and feeble; delirium.

℞. Ol. Terebinthinæ, ℥ss.

Camphoræ. gr. v.

Lac Assafœt., gr. 2 s. ft. ℥iij. ft. enema.

Also ordered Emplastrum vesicator to epigastrium, and cooling applications to head.

October 26. Epigastric pain greatly relieved by blister; mind perfectly clear; no delirium; some somnolence; tongue dry and red; pulse 83, full, soft and gaseous.

℞. Emp. Epispas. iv—iv., to nucha, to be dressed by gr. x. of Sulph. Quinia. Also,

℞. Quin. Sulph. grs. iv. q. h. ij. until four doses were taken.

October 27. Some slight delirium and some epigastric tenderness; tongue red and dry. Ordered mucilaginous drinks with infusion of cinchona.

October 28. Improving. Tongue slightly red; no cephalalgia; skin of moderate temperature; pulse 80, soft and regular.

℞. Quin. Sulph. gr. v.

Aquæ, ℥iij., Sig. ℥i. ter. in die.

October 29. Continues improving; sleeps well; has had no return of the chill since 24th inst. Pulse 72, full, soft and regular. Discontinue the Sulph. Quinia, continue the infusion of cinchona. Allowed a diet of mutton.

November 1. Appetite good; strength slowly returning. Ordered full diet, with a pint bottle of porter daily, discontinue all other treatment. Convalescence.

The patient's convalescence was slow, and fearing a relapse he was kept in the ward, with little or no treatment, excepting a careful, though good diet and occasionally tonic infusions. He was allowed to remain in the ward until the third day of December.

***Philadelphia Hospital, Blockley***, Dec. 18, 1842.

In this case we had the two distinct forms of fever which are most frequent in the United States. The geographical situation of Philadelphia is such as to render it very favourable for the comparative study of the two classes of these diseases. The town itself is placed partly on the rocks of the primitive formation, which form the falls of many of our rivers, and partly on the alluvial strata which are here met with on both shores of the Delaware. During the past season an unusual number of intermittents occurred chiefly in the alluvial formations of the southern part of the city, opposite to the Hospital; but scarcely any cases occurred there, situated, as it is, on the high gravelly ridge on the western banks of the Schuylkill. There is strong reason, indeed, for believing that the intermittent was not contracted in the Hospital itself, but resulted from the former exposure of the patient as a boatman on the Schuylkill, in which occupation he was exposed to whatever malaria might be generated upon the river—living, as he necessarily did, and sleeping on board of his boat.

During the course of the typhus fever, which was mild, though well characterized, no symptoms of the intermittent showed themselves; nor was it until convalescence was pretty well established that the chills came on. The danger of the patient was evidently vastly greater from the intermittent than from the continued fever, but as the former disease is almost always directly under the control of medicine, it was readily arrested from quinine given in high doses. As soon as the disease assumed a severe form, the quantity directed was a drachm in twenty-four hours, divided into ten doses of ten and five grains each—besides its external use. But few cases of intermittents or remittents, in our neighbourhood, require such active treatment; but now and then cases occur in which the patient's safety depends upon the early and free administration of quinine. They are sometimes not a little embarrassing, because the intermittent type of the disease is masked, as in this case, by some previous affection, or because the chill occurs at night, and the severity of the case is not at first suspected.

I have never met with a case similar to this one, in which the intermittent occurred in such close connection with the typhus fever—each disease preserving its own symptoms unchanged, unless we should attribute the unusual severity of the intermittent to the debilitating effects of the continued fever. The recovery of the patient was complete.

